# Gold Inlays

**Published:** 1907-02-15

**Authors:** C. H. Worboys


### GOLD INLAYS.

BY C. H. WORBOYS, D.D.S.

Read before the Detroit Dental Society, November 8th, 1902.

The attention of the profession today is fixed upon the making of Gold Inlays for the preservation of the natural teeth that are so badly decayed as to require large fillings to restore them to normal form and usefulness.

Some of the claims set forth for the Gold Inlay can be enumerated as follows:

To restore the form without crowning and without the necessity of devitalizing.

To relieve the patient from the long tedious operation which the malleted gold filling requires.

To avoid the danger of fracturing the tooth in the preparation of the cavity, also fracturing the tooth by expansion of the mass of gold when heated by the finishing strip.

To relieve the operator of hours of back-ache producing labor and to make a filling that is without question one of the very best, because it has only one point against it, which is its color, and this can be modified somewhat by using gold that is alloyed with silver only, making it 18K.

I will first endeavor to describe my method of making the solid gold inlay, and will take for the case a disto-occlusal cavity of a second lower molar. The cavity should be prepared by extending the buccal and lingual walls sufficiently to not require the separator for most cases that are presented. The cervical portion should be rather flat with the floor inclining slightly inward. This form gives a locking to the base of the inlay which prevents it from leaving that portion of the tooth when the stress of mastication is applied. The occlusial surface should be prepared with a step in the sulcus which will admit the inlay being displaced in one direction only. The cavity walls are prepared so that there is a slight draft, and as smooth as an excavator or chisel will cut them. I am not in favor of the cross-cut burr for the final preparation of this class of cavities, as it leaves a rough interior which is not needed for retention, but does interfere with the removal of the matrix. The margin of the cavity should be quite sharp and well defined, angular in preference to the rounded form which would leave a very thin edge to the inlay, and by not marking the matrix distinctly makes it more difficult to trim definitely when finishing outside the mouth.

With the cavity prepared, the next step is the formation of the matrix which for the solid inlay should be made of platinum. The cavity should be smeared with vaseline or soap to allow the metal to slip to place better and respond to the burnisher with greater freedom. Wet cotton is quite as good as anything to press the metal to the general form under the force of the burnisher or amalgam plugger. After being forced to the cervical portion of the cavity in such a way as to surely cover the cervical margin the metal should then be gently forced to the bottom of the step and then removed annealed and returned to the cavity for burnishing. The wrinkles must be smoothed down and then removed and such portion of the platinum as interferes with the draft of the matrix trimmed. In case the matrix is torn it is easily patched and then reswaged in the cavity.

We are ready now for the final fitting of the matrix which is best done by placing the platinum in the cavity, then slipping a thin piece of steel between the platinum and the next tooth. The platinum matrix is now filled with gum camphor which should be forced to place with a good stiff amalgam plugger. The object of using the gum camphor is to swage the matrix to a fit. The steel strip is removed first, then the matrix can be dislodged without changing its form, and it can be readily removed from the matrix by burning, if it will not readily loosen. A little pure gold can be flowed over the inside of the matrix to stiffen it and then reswaged and burnished close to the margin.

One point particularly I wish to call attention to is the possible stretching of that portion of the inlay that fills the groove and step. If this portion is filled with pure gold and under the stress of occlusion or mastication it is lengthened after the manner of drawing out a wire with a hammer or rolling, it causes the body of the inlay to be forced away from the tooth, in which case we have an imperfect filling, the same thing is more liable to occur with a filling that is built up of foil in the tooth. To prevent this I place a piece of irido-platinum wire in the groove of the matrix and flow the gold around it. This wire can be bent at an angle to give the form desired in the finished inlay. With the wire in place in the swaged matrix, the tooth side of the matrix should be painted with whiting or yellow ochre, also the cavity side, just up to the margin, after which the matrix can be filled by melting in pieces of 18 carat gold, alloyed with pure silver, which gives the gold a much lighter yellow color. When the matrix is filled to the proper contour, the platinum should be trimmed as close to the margin as possible, and then cleaned and placed in the cavity and held with a soft pine wedge while the occlusal surface is fitted. The fitting is best done before setting with the cement. After the inlay is ground and the margin properly dressed down to a fine joint it should be taken to the laboratory and given a polish that is like a mirror, after which it can be set and will not require any heavy burnishing of the edge. If you depend on burnishing the gold over the cement joint before the inlay is entirely completed you will find that the cement is hard by the time you get the inlay cut down to the tooth. For this reason finish the inlay before cementing to place.

The solid inlay is sometimes made by filling the matrix with some form of precipitated gold while it is in place in the mouth, and either 20K. plate or solder flowed over it after the inlay is removed from the mouth. This gives a very well adapted form to the matrix and for some forms of cavities is very rapid, as the rubber dam is not applied and the same care is not required in placing the gold as in the case of a filling that is built in the tooth to form a water tight plug.

I have made use of the solid gold inlay to restore that portion of the root that extends from the alveolar process to the gum margin when a piece of the root has broken away.

The scheme is this: Save the piece of tooth that has broken away, place it in softened dental lac with the broken side down so as to get an impression of that surface in the dental lac. When the material is cold. swage a piece of 1.1000 platinum in the impression with rubber. Remove and fuse in pure gold until you have the broken piece reproduced in metal This piece is now placed on the tooth root and you will find that it fits. The next step is to fit a band of gold around the inlay and root, letting it extend under the gum as far as you think best. Now grasp the inlay and band with a pair of pliers and remove both together and unite the two with solder. After soldering finish as smoothly as possible and set as you would a crown. You may now do as you wish with the top of the root, as it is nearly as good as before. I can report but two cases of this kind, but am pleased to say that they are doing good service and are in fine condition.

There are two ways of constructing the hollow inlay, the swaged, and the filled over the cement or an investing compound.

I will first describe the swaged hollow inlay, which is indicated where there is much restoration of the tooth substance necessary. The cavity preparation is the same as for the solid inlay, that is it should have a slight draft, and if possible be formed so that it can be displaced only in one direction. Pure gold of about 30 gauge is burnished to fit the cavity as closely as possible, and the margin well defined. When this is well done the gold should be trimmed almost to the margin, carefully annealed and then replaced in the cavity and swaged to place with rubber, cotton, or anything you may use for the purpose.

Now remove and *very* carefully anneal and replace in the cavity. A small piece of softened modeling compound is now placed in the gold matrix and the patient told to bite it hard and hold till cold. In case of a cavity with an approximal wall gone, a thin piece of steel should be placed between the adjoining tooth and compound, and when the latter is cold remove the steel which will allow the compound and gold matrix to be easily removed. The compound should now be carved to just the form the finished filling is to be, *less* the thickness of the gold plate that is to be swaged over it. The gold matrix with the modeling compound in place is now placed in the swager invested with dental lac in such a way that the margin is just exposed, but supported well all around. A piece of pure gold plate a little larger than the size necessary to lap the margin should be bent with a plier in such a way that it somewhat conforms to the modeling compound, and then annealed and place in position and swaged. After swaging, the piece should be gently removed and next the modeling compound taken from the matrix which should be left in place in the lac, while a hole about 1-16 inch is bored through it at a point about equal distance from the margins.

The matrix should be carefully removed from the lac and placed on the swaged contour and the two pieces should be held in something that will support the whole piece, because the thin gold will not hold up when heated to redness. Care must be taken in soldering the two pieces together, first, that very little borax be placed inside or the pieces will be separated before the borax glasses; second, that the solder be placed inside through the drilled hole about where it is desired, and enough of it to make the grinding surface of the inlay sufficiently strong to withstand the stress of mastication, and third, that the margin be protected by whiting from the solder flowing out on the gold that has been adapted to the tooth.

After the case is soldered it is ready for finishing. If the margin has been well defined the gold can be trimmed with the shears and a paper disk will do the rest.

When finished and ready to set in the tooth, the inlay should be nearly filled with cement that has been mixed stiff enough to be well packed, as it is almost impossible to fill the inlay with the cement that it should be set with.

Another form of hollow gold inlay is made by partially filling the gold matrix with cement or some quick setting investing compound, and then building the contour with some form of the precipitated gold either in the mouth with hand pluggers or out of the mouth with rubber swager, and over the gold flow 20 carat solder or plate. After finishing, a hole is bored through the matrix, and the lining removed and then filled with cement and set.

A cone burr with the point ground off makes a very convenient instrument to give the proper draft to a cavity when it can be used.

### DISCUSSION.

DR. F. W. MACDONALD: The essayist has not detailed all the methods, but he certainly has given us two good methods. We all want the simplest as well as the best method. We can use the hollow inlay many times to better advantage than the solid, although the latter may be easier to make and will stand more stress. The hollow inlay, if fitted with cement, is preferable, because it does not convey temperatures so readily. I don't agree with essayist in his cavity preparation. I would cut the cervical floor of the cavity on a right angle, or even at an obtuse angle to the surface of the tooth, rather than to cut it so that it will dip down from the cavity margin to the pulpal wall. I rather get retention here by slightly grooving with a No. 3 round burr. It will be more difficult to withdraw the matrix from the essayists cavity form.

I don't see any use in the iridio-platinum wire the essayist places in the retention arm on the crown surface. I don't believe the stress of mastication is sufficient to dislodge the inlay by stretching this arm.

I don't believe it necessary to sacrifice good sound tooth structure to extend the cavity margins so that the matrix can be more easily removed. I believe it better to separate the teeth, or use cement to fill in the undercuts. I prefer slow separation, and like plenty of space.

I have good success in swaging matrices. I prepare the cavity and get impression at first sitting, and then finish matrix by burnishing at the next sitting, when I make and set the inlay. This is easier for patient. I use a metal ring, similar to the Brophy matrix, which I slip over the tooth, and put the soft modeling compound into the cavity. When it is cool I slip the ring and all from the tooth and fill the impression with Ame's Crown and Bridge Cement, which will set hard enough to swage the matrix into the crown swager. You can even burnish on the delicate cavity margins of such a die. The matrix is finished by burnishing in the tooth cavity. The matrix should be filled to contour with wax and Hinman's matrix metal adapted to the surface. The wax is then melted out and the edges are soldered. The advantage of a gold inlay is that a better finish can be made on the surface, so that it can be kept cleaner, and it avoids the strain on the patient of the long operations of gold fillings in large cavities.

I have made solid inlays successfully for the incisal surfaces of lower front teeth. I make the cavity box shape, with a pit at either end, into which I project pins soldered to the matrix. I use platinum pins taken from old porcelain teeth. These pins will securely hold the matrix when the anchorage of a gold filling would be difficult. I usually fill these matrices with solder, but they may be contoured at will with mat gold.

In cementing inlays I have used Ames oxyphosphate of copper at the cervical portion of the cavity and the oxyphosphate in the visible portion of the cavity to prevent absorption of the cement at the cervical border.

Dr. A. L. LeGro: The gold inlay, in my judgment, is more than a passing fad. It has come to stay, and while it will never supplant other filling methods, it has a sphere of usefulness that makes it a most valuable method. Many teeth can be better filled with inlays than by any other process. The enthusiast generally discourages the average practitioners, because he claims too much, The gold inlay is not an easy operation to make, and anyone who thinks so had better let it alone, as he will fail and be disappointed. The cavity preparation must be done with exactness, and the technique requires skill and patience.

I prefer the pure gold inlay, and make it solid. If you have to use stiff cement in setting the hollow inlay you are no better off than if you had made it hollow, with the exception that perhaps there is less conduction and irritation of the dentine. I like the pure gold inlay because the margins are soft, and can be burnished to close adaptation. I prefer the burnished matrix in all cases as I can get closer adaptation. I fill the matrix with pure gold, when the matrix is fitted remove from the cavity, trim to margins, and flow enough gold over the bottom to stiffen it, then replace in the cavity and reburnish, and flow more gold by sweating the gold a little at a time until the desired contour is attained. When the inlay is complete use a brush flame of the blowpipe to smooth the entire surface. It is then ready to polish and set.

Dr. M. L. Ward: The technique of the gold inlay is more difficult than most writers and speakers lead us to think. It is one of the most difficult operations to make and secure desirable results. Some men are so skillful that, like Dr. Peso, they can take a gold matrix on a soldering block and sweat it full of pure gold, but few of us can do that. I have succeeded by placing the matrix in a nickle tray filled with silex. I don't trim the borders as Dr. LeGro suggests until the matrix is filled. I use the platinum matrix and fill it with pure gold. Pure gold may make a clearer inlay, but it changes form so easily in soldering. The idea of the essayist that the gold arm used for retention in the crown of the tooth will stretch under mastication, is more theoretical than natural I think.

DR. G. C. BOWLES: I am almost ready to say that the gold inlay is more useful to me than the gold filling. I make the hollow inlay by burnishing the matrix to place and then soldering No. 36 plate for the proximal wall, and then tack on a piece of the same plate for the occlusal surface, and place in the cavity, and have the patient occlude the teeth while I form and burnish it to the desired shape, it is then removed and soldered as desired. If the borders are left full the soldering is accomplished more easily, and the finishing can be done with equal exactness.

DR. WORBOYS: I have seen many good gold fillings that were certainly crowded out of the cavity, or at least away from the borders so that the cavity was opened up for decay producing agents, because the crown or incisal retention had been battered by the impacts of mastication so as to elongate it and force the body of the filling away from its seat in the tooth. Gold is a soft metal, and when used pure and in so small amount the chances are that it will change form under heavy impacts. Why then take chances with it when we can make secure work with no great trouble or expense?

I don't believe any one can burnish a matrix to fit a cavity —at least it is not nearly so accurate a method as the swaging. You can burnish to form, but not to fit. If you will try to burnish a piece of pure gold plate to a flat surface you will soon see how difficult a thing it is to do. You can get much better results by burnishing the matrix to place approximately, and completing it by swaging with gum camphor.

The hollow matrix must be filled solid with a stiff cement or it will dent or cave in under the stress of mastication, it is too frail for most places where strength is required. If you use the method described by Dr. Bowles, of having the patient bite the soft gold into form, you should have some yielding material, like wax, inside of the crown, or your whole inlay will be distorted. The final fitting of inlays should be done before cementing to place, and all exposed surfaces should be polished as smooth as possible before setting.

